# Sense of achievement

**DOI:** 10.7554/eLife.01605

**Published:** 2013-10-15

**Authors:** Markus Knaden, Bill S Hansson

**Affiliations:** 1 **Markus Knaden** is at the Max Planck Institute for Chemical Ecology, Jena, Germanymknaden@ice.mpg.de; 2 **Bill Hansson** is at the Max Planck Institute for Chemical Ecology, Jena, Germanyhansson@ice.mpg.de

**Keywords:** Odorant receptors, antenna, electrophysiology, cheminformatics, *D. melanogaster*

## Abstract

Computational techniques developed to predict if odorants will interact with receptors in the olfactory system have achieved a success rate of 70%.

**Related research article** Boyle SM, McInally S, Ray A. 2013. Expanding the olfactory code by in silico decoding of odor-receptor chemical space. *eLife* **2**:e01120. doi: 10.7554/eLife.01120

**Image** Network connecting odorant receptors (large circles) and the compounds (black dots) that they are known (purple lines) and predicted (grey lines) to be sensitive to

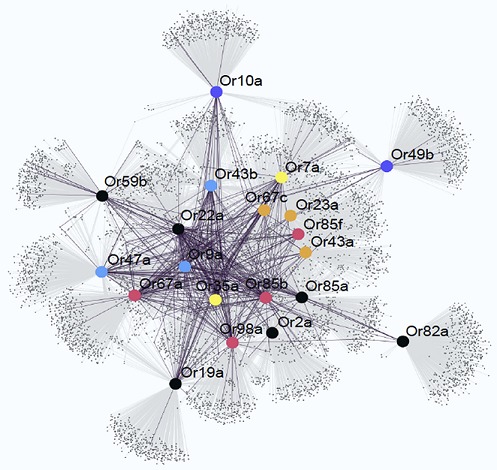

The question of how we and other animals perceive the surrounding world was tackled by Aristotle more than 2300 years ago. Since then we have gained quite a good understanding of visual perception. Humans and most other animals employ a small number of different types of visual receptor, each being most sensitive to light of a specific wavelength and less sensitive to shorter or longer wavelengths (Schnapf et al., 1987). Using different receptor types, with overlapping sensitivity ranges, we can detect light with wavelengths between about 380 nm and 750 nm. Hearing is also well understood: sounds of different wavelengths activate different types of sensory neurons to provide coverage over a range of wavelengths (Masterto et al., 1969). However, the way that we respond to our chemical environment—that is, the way we respond to different smells and tastes—is much more complicated.

Contrary to vision and audition, olfaction has to deal with cues that are not arranged along a linear scale. The nose is exposed to several hundred thousand odorants that differ in chemical structure and in ecological relevance. One might imagine that the nose would need numerous different receptor types—each type sensitive to just a single compound—to detect and discriminate all the relevant odorants. However, as always, evolution found a smarter way. As first discovered by Richard Axel and Linda Buck in 1991—and rewarded with a Nobel Prize in 2004—animals are equipped with a relatively small, species specific, number of olfactory receptors (Buck and Axel, 1991): mice have more than 900 types, humans about 400, and the vinegar fly *D. melanogaster* has around 60.

Only a few, very important odorants—such as pheromones (Nakagawa et al., 2005) or the odorants given off by rotten food (Stensmyr et al., 2012)—have a one-to-one relationship with specific olfactory receptors. In general, a single receptor can detect a range of different odorants, and a single odorant can target a range of receptors, with a given odorant being identified through the pattern of receptors that it activates (Hallem and Carlson, 2006). It is thought that this so-called combinatorial olfactory code is employed by insects and also by vertebrates (Vosshall, 2000; Kauer and White, 2001). However, many of the details of the interactions between the odorant molecules and the receptors remain mysterious. Why, for example, do odorants with similar structures sometimes target different receptors, whereas other odorants with clearly different structures often target the same receptor.

Now, in *eLife*, Sean Boyle, Shane McInally and Anandasankar Ray of the University of California at Riverside describe a new method that can predict which odorants interact with which receptors much more accurately than previous methods (Boyle et al., 2013). During the last decade many groups have screened the sensory range of the odorant receptors of the vinegar fly, and a total of 251 different odorants are known to be able to activate at least one receptor. Although this is a tiny number compared with the number of odorants that flies are usually exposed to, Boyle, McInally and Ray were able to gain fresh insights into the receptor-odorant interactions by performing a highly detailed meta-analysis on these 251 odorants to identify the properties that cause an odorant to target a particular receptor (Figure 1). In addition to the ‘usual suspects’ of molecular properties (e.g., whether the odorant is an alcohol, an ester or an aldehyde), they took into account some 3,224 physical and/or chemical properties of the odorants, including obvious properties like molecular weight and three-dimensional structure, and less obvious properties like the ‘eigenvalue sum from electronegativity weighted distance matrix’.

**Figure 1.**
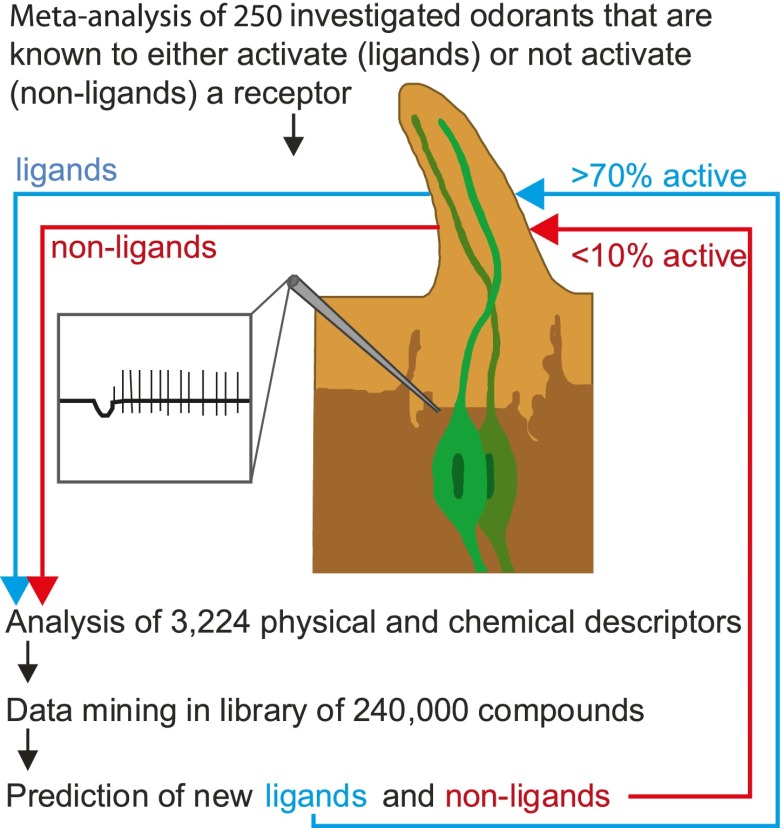
Predicting odorant-receptor interactions. Boyle et al. performed a meta-analysis of 250 odorants and 51 receptors and developed an algorithm (based on some 3,224 physical and chemical properties of the odorants) to predict whether a given odorant will interact with a given receptor. This algorithm was then used to ‘mine’ a library of 240,000 compounds and identify ligands (blue line) and non-ligands (red line) for nine receptors. Experiments were performed with 141 compounds (11–23 per receptor): 71% of the compounds that were predicted to be ligands were found to interact with the relevant receptor, and less than 10% of the compounds that were predicted to be non-ligands were found to interact. The illustration shows an insect sensillum housing two olfactory receptor neurons (one pale green, the other dark green), each with a cell body and a nucleus, and a dendrite that extends into the tip of the sensillum. The tip is filled with a fluid called the sensillum lymph (pale brown) that is excreted by trichogen cells (dark brown). The expanded detail shows the neuronal response to a ligand as measured in the single sensillum recordings performed by Boyle et al.

This approach was pioneered by groups at Goethe University in Frankfurt (Schmuker et al., 2007) and the Weizmann Institute (Haddad et al., 2008). However, instead of analysing all the receptors and all the physical and chemical properties, the Riverside researchers used an algorithm that allowed the most critical properties for each receptor to be identified. Next they screened a list of more of 240,000 odorants to find those that they expected to interact with nine different receptors. Finally, they tested these predictions in experiments: Their predictions were correct more than 70% of the time, compared with a success rate of just 10% for odorants chosen at random. Hence, although odorants do not follow any linear rules like light and sound, we can still use their physical and chemical properties to predict whether an odorant interacts with a specific receptor and later, we hope, be able to understand why it interacts.

These results will be of interest beyond a narrow group of specialists. According to the United Nations Food and Agriculture Organization, insects and insect-spread diseases are responsible for an estimated 20–40% of world-wide crop production being lost every year. Furthermore, malaria and dengue fever, which are both spread by mosquitoes, kill more than 1 million people every year (and infect another 250 million). As insects typically use olfactory cues to find new hosts, a better understanding of odorant-receptor interactions promises substantial improvements for human food supply and health.
